# Partial depletion of CD206-positive M2-like macrophages induces proliferation of beige progenitors and enhances browning after cold stimulation

**DOI:** 10.1038/s41598-018-32803-6

**Published:** 2018-10-01

**Authors:** Yoshiko Igarashi, Allah Nawaz, Tomonobu Kado, Muhammad Bilal, Takahide Kuwano, Seiji Yamamoto, Masakiyo Sasahara, Xu Jiuxiang, Akiko Inujima, Keiichi Koizumi, Johji Imura, Naotoshi Shibahara, Isao Usui, Shiho Fujisaka, Kazuyuki Tobe

**Affiliations:** 1 First Department of Internal Medicine, University of Toyama, Toyama, 930-0194 Japan; 2 Department of Pathology, University of Toyama, Toyama, 930-0194 Japan; 3 Division of Kampo Diagnostics, Institute of Natural Medicine, University of Toyama, Toyama, 930-0194 Japan; 4 Department of Diagnostic Pathology, University of Toyama, Toyama, 930-0194 Japan; 5 Department of Metabolism and Nutrition, University of Toyama, Toyama, 930-0194 Japan; 6 JSPS International Research Fellow, Department of Metabolism and Nutrition, University of Toyama, Toyama, 930-0194 Japan; 7 Department of Endocrinology and Metabolism, Dokkyo Medical University, Tochigi, 321-0293 Japan

## Abstract

Beige adipocytes are an inducible form of thermogenic adipocytes that become interspersed within white adipose tissue (WAT) depots in response to cold exposure. Previous studies have shown that type 2 cytokines and M2 macrophages induce cold-induced browning in inguinal WAT (ingWAT) by producing catecholamines. Exactly how the conditional and partial depletion of CD206^+^ M2-like macrophages regulates the cold-induced browning of ingWAT, however, remains unknown. We examined the role of CD206^+^ M2-like macrophages in the cold-induced browning of WAT using genetically engineered CD206DTR mice, in which CD206^+^ M2-like macrophages were conditionally depleted. The partial depletion of CD206^+^ M2-like enhanced UCP1 expression in ingWAT, as shown by immunostaining, and also upregulated the expression of *Ucp1* and other browning-related marker genes in ingWAT after cold exposure. A flow cytometry analysis showed that the partial depletion of CD206^+^ M2-like macrophages caused an increase in the number of beige progenitors in ingWAT in response to cold. Thus, we concluded that CD206^+^ M2-like macrophages inhibit the proliferation of beige progenitors and that the partial depletion of CD206^+^ M2-like macrophages releases this inhibition, thereby enhancing browning and insulin sensitivity.

## Introduction

Recent advances in our understanding of adipose tissue suggest that adipose tissue is not merely an energy reservoir but also an important component of the glucose metabolism-regulating system through its release of hormones called adipokines. White adipose tissue (WAT) stores lipids during times of caloric excess and efficiently releases fatty acids during prolonged starvation. Mammals have two other types of adipocytes, brown adipose tissue (BAT) and beige adipocytes, both of which are thermogenic and dissipate energy in the form of heat. Recently, the mechanisms controlling the functions of WAT, BAT, and beige adipocytes have been intensively studied as part of efforts to ameliorate obesity and insulin resistance^1,2^.

Macrophages represent a major stromal cell population and are considered to assist the metabolic functions of WAT, BAT and beige adipocytes^3^. The metabolic functions of WAT are greatly affected by adipose tissue-resident macrophages (ATMs), which can be classified into proinflammatory (M1) and anti-inflammatory (M2) macrophages^4–6^. We and others have shown that in lean states, the majority of macrophages in WAT are M2-like macrophages; in obese states, however, the majority of macrophages that infiltrate WAT are M1-like macrophages^4–9^. These adipose tissue-infiltrating M1-like macrophages are responsible for a persistent low-grade inflammatory state that underlies the systemic insulin resistance observed in obesity. Genes that are predominantly expressed in M2 macrophages in lean states include *Cd206, Arg1*, and *Il10*, while those expressed in M1 macrophages in obese states include *Cd11c, Il6, Tnfa*, and *Mcp1*. M2-like macrophages are reportedly involved in tissue remodeling processes, including dead adipocyte clearance and progenitor activation^10,11^. Previously, we reported that almost all CD206^+^ cells in the adipose tissue of lean mice are F4/80^+^ macrophages and that F4/80^+^CD206^+^ fractions showed higher expression of M2 marker genes including *Cd163*, *Mgl2*, etc., showing that Cd206 is a specific marker for adipose tissue-resident M2-like macrophages^12^.

Within WAT, especially inguinal WAT (ingWAT), reside clusters of thermogenic adipocytes called beige adipocytes that can be induced in response to cold exposure^13,14^. Several pieces of evidence suggest that the induction of beige adipocytes is a key factor in combating insulin resistance and obesity^15,16^. Previous reports have shown that M2-like macrophages also play a key role in the induction of browning in ingWAT *via* the activation of type 2 cytokine production during cold exposure^17–20^ and other stimuli that activate the sympathetic nervous system^10^. However, whether these mechanisms are catecholamine-dependent^17,18,21^ or catecholamine-independent^22,23^ remains a matter of debate. Macrophages, which are not necessarily M2-like macrophages, reportedly regulate catecholamine catabolism; thus, the depletion of macrophages may regulate the beige adipocyte phenomenon^24,25^. Another unresolved question about beige adipocytes concerns their origin, specifically whether they are produced through the transdifferentiation of white adipocytes into brown adipocytes^26–29^ or *via* the activation of beige progenitors^30–33^. We previously reported that a partial but specific depletion of CD206^+^ M2-like macrophages in adult mice induced the proliferation of white adipocyte progenitors and improved glucose metabolism^12^. Thus, we presumed that the specific but partial depletion of CD206^+^ M2-like macrophages might stimulate the proliferation of beige progenitors. In the present study, we examined the effect of the partial depletion of CD206^+^ M2-like macrophages on the browning of ingWAT in response to cold using previously generated genetically engineered CD206DTR transgenic mice^12^.

## Materials and Methods

### Materials

Diphtheria toxin (cat# D0564) and collagenase (cat# C6885) were purchased from Sigma-Aldrich. The RNeasy Mini Kit (cat# 7404 and 74106) was purchased from Qiagen. The PCR primers used with the TaqMan method were purchased from Applied Biosystems, while those used with the SYBR Green method were purchased from Invitrogen^TM^ Life Technologies, Japan. The SYBR Green primer sequences are available upon request.

For the flow cytometry analysis, all the reagents and antibodies including the PE hamster anti-mouse CD11c (cat# 553802) and 7-amino-actinomycin D [7AAD] (cat# 559925) antibodies were obtained from BD Biosciences. PE hamster IgG, a polyclonal isotype control (cat# ab32662) antibody was purchased from Abcam. APC/Cy7 anti-mouse F4/80 (cat# 123118) and APC/Cy7 rat IgG2a, κ isotype control (cat# 400523) antibodies were obtained from BioLegend. Rat anti-mouse CD206 conjugated Alexa Fluor 647 antibody (cat# MCA2235A647) and rat IgG2a Alexa Fluor 647 negative control antibody (cat# MCA1212A647) were obtained from AbD Serotec. PE anti-mouse CD137 (cat# 106106) and PE rat IgG2a, κ isotype control, antibodies were purchased from BioLegend. PE/Cy7 anti-mouse CD45 (cat# 25-0451-82), PE/Cy7 anti-mouse CD31 (cat# 25-0311-81), and PE/Cy7 rat IgG2a, κ isotype control (cat# 25-4321-81) antibodies and purified anti-mouse CD16/CD32 (cat# 14-0161-86) were purchased from eBioscience.

For immunohistochemistry, anti-rabbit UCP1 (cat# ab10983) and anti-rabbit Ki-67 (cat# ab15580) antibodies were purchased from Abcam. Rat anti-mouse FITC CD137 (cat# 558975) antibody was purchased from BD Biosciences. Anti-rabbit IgG Fab2 Alexa Fluor 555 (cat# 4413S) secondary antibody was purchased from Cell Signaling.

### Methods

#### Animals and cold exposure

Male C57BL/6J mice were purchased from Jackson Laboratory and were housed under an alternating dark/light cycle in the animal care facility of the University of Toyama, Toyama, Japan. To clarify the role of CD206^+^ M2-like macrophages, we used genetically engineered CD206DTR transgenic (Tg) mice based on the transgenic expression of the diphtheria toxin receptor (DTR) under the control of the CD206 promoter to specifically ablate CD206^+^ M2-like macrophages^12^ (Fig. S1A,B). Tg founders were then once again backcrossed to C57BL/6 J mice. The male F4 generations and beyond were used for the experiments to derive the data. Wild-type (WT) littermates were used as controls in all the experiments. Diphtheria toxin (DT) injection was used to partially but specifically deplete CD206^+^ M2-like macrophages. Three DT injections at a dose of 0.003 mg/kg body weight were sufficient for the partial depletion of CD206^+^ M2-like macrophages. All the mice were fed a normal chow diet and had free access to water. For cold stimulation, the mice were individually placed in conventional cages (one mouse per cage) and then subjected to cold (6 °C) for 48 h, 72 h or 96 h. Control mice were individually placed in conventional cages at room temperature (RT) (24 °C) for the same durations. DT-administered WT and Tg mice were also placed in cold chambers for 96 h, while the control groups (DT-administered WT and Tg mice) were placed in cages at RT for 96 h. The dietary and light conditions were the same for both groups throughout the study. Body weight and food intake in both the RT and cold groups were measured daily. All the experiments were performed in accordance with the relevant guidelines and regulations and were approved by the Committee for Institutional Animal Care and Use of the University of Toyama (Toyama, Japan).

#### Genotyping

Whole genomic DNA was derived from the tail and then lysed in DirectPCR (tail) lysing solution (cat# 102-T) from Viagen Biotech and recombinant PCR grade Proteinase K (cat# 03115828001) from Roche Diagnostics, Germany, according to the manufacturer’s instructions. This crude DNA was then used for PCR using the Tks Gflex DNA Polymerase kit (cat# R060A) from TaKaRa, according to the manufacturer’s instructions and using a previously described method^12^.

#### Histology

Brown adipose tissue (BAT) and inguinal WAT (ingWAT) samples were fixed in 4% paraformaldehyde (PFA), dehydrated, and embedded in paraffin wax. The paraffin-embedded tissues were cut into 5-μm-thick sections and mounted on slides. The sections were then stained with primary and secondary antibodies according to the manufacturers’ instructions. Data were collected using H&E-stained sections from 4–6 mice in each group (20× and 40× magnifications). UCP1 immunohistochemistry was performed using a polyclonal anti-UCP-1 antibody (1:50). The sections were examined by microscopy (Olympus BX61/DP70). An immunofluorescence analysis of ingWAT was performed using anti-CD137 (1:100) and anti-Ki-67 (1:100) antibodies as well as anti-CD137 (1:100) and anti-UCP1 (1:50) antibodies. All the primary antibodies were incubated overnight at 4 °C. All the sections were incubated with the relevant secondary antibodies (1:250) and DAPI (1:500) for 2 h in the dark at room temperature. All the micrographs were obtained using a TCS SP5 Leica confocal microscope (40× magnification).

#### Real time quantitative polymerase chain reaction (RT-qPCR or qPCR)

Total RNA was isolated from BAT and ingWAT using the RNeasy® mini kit (Qiagen, Germany), and the cDNA synthesis and qPCR analysis were performed as described previously^8,9,34,35^.

#### Physiological analysis

Intraperitoneal glucose tolerance and insulin tolerance tests (IP-GTT and IP-ITT, respectively) were performed as described previously^12,35^.

#### Flow cytometry analysis

Isolation and separation of the stromal vascular fraction (SVF) and subsequent flow cytometry were performed as previously described^8,12,35^. After the exclusion of dead cells by gating with 7-amino-actinomyciin D (7AAD), live cells in the SVF of ingWAT were selected for further analysis. First, CD31/CD45^+^ (endothelial/hematopoietic) cells were negatively selected (1:100), followed by the positive selection of CD137^+^ cells (1:80). For CD206^+^ M2-like macrophages, after removing debris, live cells were gated for CD45^+^ (hematopoietic), and then CD45^+^ cells were gated for the F4/80^+^CD206^+^ double positive population, which was characterized as consisting of M2-like macrophages. An isotype control antibody was used as a negative control. The experiments were performed using a FACS Diva Version 6.1.2 automated cell analyzer (BD FACS Canto II).

#### Statistical analysis

Data are expressed as the mean ± SEM. Statistical significance was determined by unpaired Student’s t tests (for comparisons of two groups or two experimental conditions). Differences were considered statistically significant at values of *p < 0.05 or **p < 0.01.

### Ethical standard

All institutional and national guidelines for the care and use of laboratory animals were followed.

## Results

### Effect of cold stimulation on BAT and WAT

Similar to previous reports^36–38^, we found that 96 h of cold stimulation changed the morphology and mass of both BAT and ingWAT (Fig. S2A–G). Gene expression and immunohistochemical analysis confirmed that upon exposure to cold, browning is induced in ingWAT (Fig. S3A–C). Taken together, these results confirm that cold stimulation enhances browning in ingWAT of WT mice.

### Effects of cold stimulation on ingWAT of WT and Tg mice

**Figure 1:**
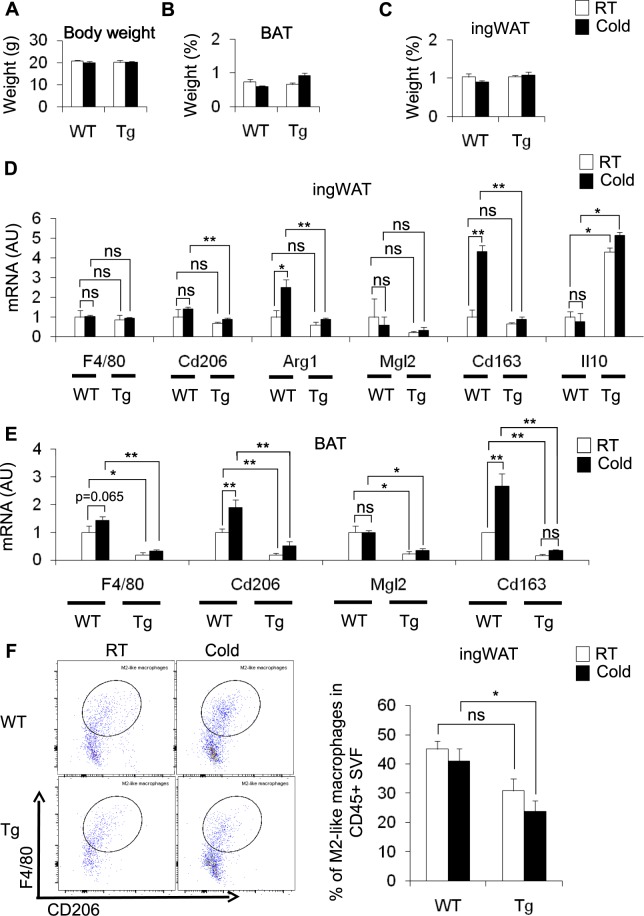
Effect of cold stimulation on BAT and ingWAT in WT mice and DT-treated CD206DTR transgenic (Tg) mice. (**A**–**C**) Changes in body weight (g) and adipose tissue weight (BAT and ingWAT) of WT and Tg mice at RT (white) and at a cold temperature (black) as the percentage of body weight (n = 4–6 mice per group). (**D**,**E**) mRNA expression of M2-like macrophage marker genes in the ingWAT and BAT of cold-stimulated WT and Tg mice compared with their littermate controls at RT (n = 3–5 mice per group). (**F**) Representative flow cytometric images of ingWAT from WT and Tg mice (RT vs. cold) (left panel) showing M2-like macrophages. The M2-like macrophages in CD45^+^ SVF are quantified in the right panel. After debris removal, live cells were gated for CD45^+^ cells (hematopoietic) and then for F4/80^+^CD206^+^ cells (M2-like macrophages) (n = 3–4 mice per group). An isotype control antibody was used as a negative control. The data are shown as the mean ± SEM. *p < 0.05, **p < 0.01, compared with their littermates, as determined using Student’s *t*-test (AU: arbitrary unit, ns: non-significant).

**Figure 2:**
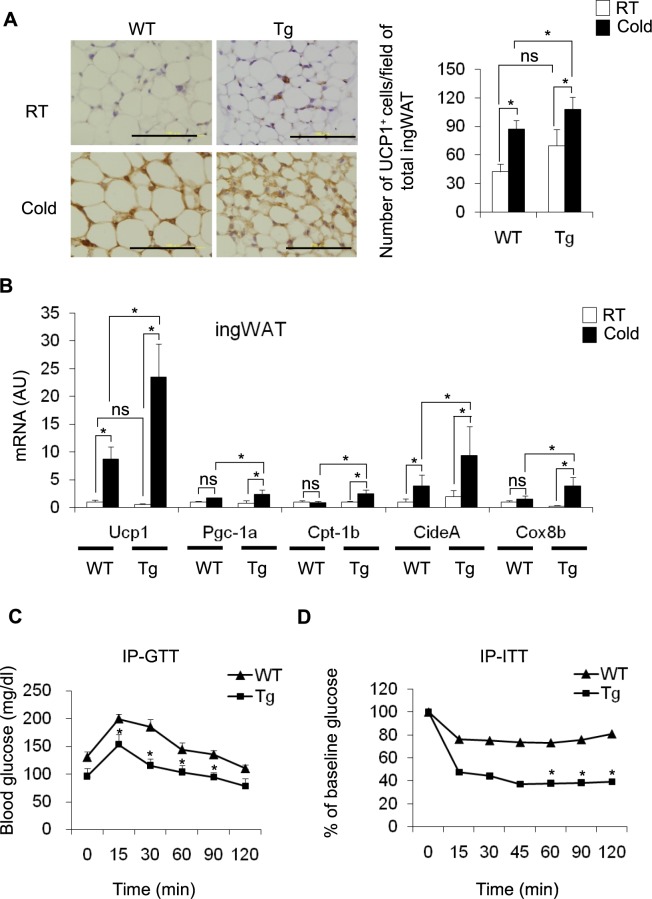
Partial depletion of CD206^+^ M2-like macrophages increases browning of ingWAT. (**A**) Representative images of paraffin sections of ingWAT from DT-treated WT and Tg mice after cold exposure (96 h) stained with anti-UCP1 antibody (left panel). UCP1^+^ cells were counted in 5 randomly selected microscopic fields (20×) and were expressed as the average cell number per microscopic field/total ingWAT weight (right panel) (n = 4–6 mice per group; scale bar, 100 μm). (**B**) mRNA expression of *Ucp1* and other browning marker genes in the ingWAT of cold stimulated DT-treated WT and Tg mice (n = 4–6 mice per group). (**C**,**D**) Glucose concentrations from an intraperitoneal glucose tolerance test (IP-GTT) (n = 7 mice per group) and glucose concentrations from an intraperitoneal insulin tolerance test (IP-ITT) as the percent of the basal glucose level (n = 7–9 mice per group) in DT-treated WT and Tg mice maintained at a cold temperature. The data are shown as the mean ± SEM. *p < 0.05, **p < 0.01, compared with littermates, as determined using Student’s *t*-test.

To investigate the role of CD206^+^ M2-like macrophages in the induction of browning in ingWAT, we used genetically engineered Tg mice in which CD206^+^ M2-like macrophages can be partially depleted after diphtheria toxin (DT) administration. We administered DT at a dose of 0.003 mg/kg body weight to both WT and Tg mice on three alternate days to partially deplete CD206^+^ M2-like macrophages (Fig. S4A). Under cold stimulation for 96 h, food consumption and body weight remained unchanged in both the DT-treated Tg and control DT-treated WT mice (Fig. S4B and Fig. 1A). In addition, the BAT and ingWAT weights in the cold stimulated DT-treated Tg mice were not different than those of the cold stimulated DT-treated WT mice (Fig. 1B,C). Then, we examined the effect of DT treatment on the expression of M2 and M1 marker genes in the ingWAT (Fig. 1D and Fig. S4C) and BAT (Fig. 1E and Fig. S4D). DT treatment decreased the expression of M2 macrophage marker genes including *Cd206 (Mrc1), Arg1*, and *Cd163* in ingWAT of Tg mice both at RT and in the cold, indicating that M2-like macrophages were successfully depleted, albeit partially (Fig. 1D). Interestingly, the expression of *Il10* was increased in Tg mice compared with their littermate controls at RT and cold temperature (Fig. 1D). Similarly, reduced expression of M2-like macrophage markers (*Cd206, Mgl2*, and *Cd163*) was observed in the BAT of DT-treated Tg mice (Fig. 1E). We next examined the expression of M1 macrophage marker genes in the ingWAT (Fig. S4C) and BAT (Fig. S4D) of DT-treated Tg mice and their littermate controls. We found that the expression of M1-like macrophage marker genes including *Tnfa, Il1b, Mcp1* and *Cd11c* were upregulated in the ingWAT of DT-treated Tg mice compared with their littermate controls at RT and at the cold temperature. Interestingly, cold stimulation increased the expression of *Nos2* without affecting the expression of other M1 markers (Fig. S4C). In contrast to ingWAT, the expression of M1-like marker genes including *Tnfa, Mcp1* and *Nos2* was significantly reduced in the BAT after partial depletion of CD206^+^ M2-like macrophages in Tg mice (Fig. S4D). A flow cytometry analysis further showed that the percentage of M2-like macrophages (F4/80^+^CD206^+^) in the CD45^+^ fraction of ingWAT SVF was significantly decreased in DT-injected Tg mice compared with DT-administered WT mice maintained at both cold temperatures and RT (Fig. 1F and Fig. S4E). However, the percentage of M2-like macrophages in the BAT was not different between DT-administered WT and Tg mice maintained at either the cold temperature or RT (Fig. S5). A morphological analysis also revealed that the size of ingWAT was not altered upon cold stimulation in DT-treated Tg mice compared with DT-treated Tg control mice at RT and DT-treated WT mice at both cold temperatures and RT (Fig. S6). An immunohistochemical analysis of ingWAT stained with anti-UPC1 antibody also showed an increase in the number of UCP1^+^ cells in the ingWAT of cold-induced DT-treated Tg mice compared with that in WT control mice (Fig. 2A), showing that browning is increased in the ingWAT. Consistent with this finding, a gene expression analysis revealed that *Ucp1* expression was upregulated together with other browning marker genes in the ingWAT of DT-treated Tg mice (Fig. 2B). Interestingly, we further found that the partial depletion of CD206^+^ M2-like macrophages improved glucose tolerance and enhanced insulin sensitivity in cold-stimulated Tg mice compared with that in their WT littermates (Fig. 2C,D). These results indicate that the partial depletion of CD206^+^ M2-like macrophages promotes the browning of ingWAT in response to cold, thereby inducing insulin sensitivity.

### Effect of cold stimulation on the proliferation of beige progenitors in the ingWAT of CD206^+^ M2-like macrophages depleted mice

**Figure 3:**
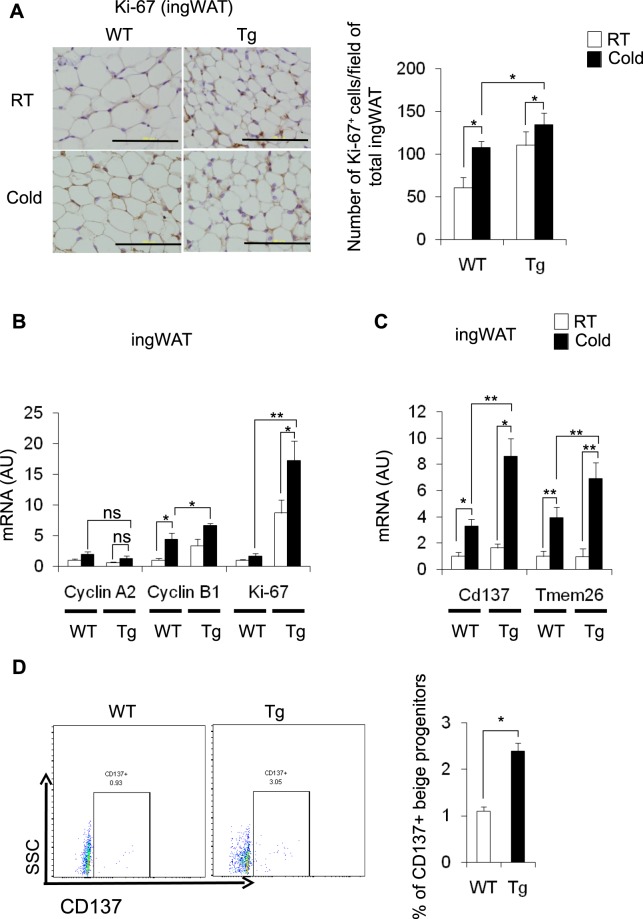
Effect of cold exposure on cell cycle-related genes and beige progenitor genes in ingWAT of cold-stimulated Tg mice. (**A**) Representative images of paraffin sections of ingWAT of DT-treated WT and Tg mice after cold exposure stained with anti-Ki-67 antibody (left panel) and quantification of Ki-67^+^-cells/field for total ingWAT weight (right panel) (n = 4–6 mice per group; scale bar, 100 μm). **(B**,**C)** mRNA expression of cell cycle-related genes and beige progenitor marker genes in the ingWAT of cold-stimulated WT and Tg mice compared with their littermate controls at RT (n = 4–6 mice per group). (**D**) Representative flow cytometry images of ingWAT from WT and Tg mice (left panel). Quantification is shown in the right panel. After debris removal, live cells were gated for CD31/CD45^−^ cells (lineage negative). Then, the lineage negative cells were further gated for the CD137^+^ population (n = 4–5 mice per group). An isotype control antibody was used as a negative control. The data are shown as the mean ± SEM. *p < 0.05, **p < 0.01, compared with littermates, as determined using Student’s *t*-test.

**Figure 4:**
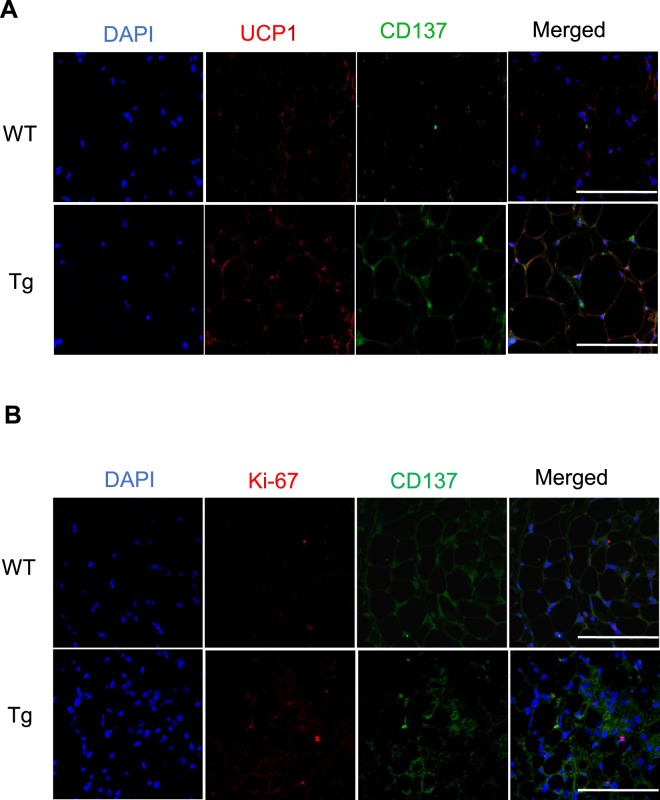
Immunofluorescence analysis of ingWAT. (**A**,**B**) Representative confocal images of paraffin sections of ingWAT from DT-treated WT and Tg mice after cold exposure stained with anti-UCP1 and anti-CD137 antibodies (**A**) and anti-CD137 & anti-Ki-67 antibodies. (**B**) The pictures were taken using a Leica TCS-SP-5, 40× (scale bar, 50 μm).

We examined whether the partial depletion of CD206^+^ M2-like macrophages affected the proliferation of beige progenitors. An immunohistochemical analysis revealed that the number of Ki-67^+^ cells was increased in DT-treated Tg mice upon cold exposure compared with the level in control Tg mice under cold stimulation (Fig. 3A), indicating an increase in proliferating cells in the ingWAT of DT-treated Tg mice. Consistent with this finding, we observed upregulated expression of cell cycle-related genes in the ingWAT of DT-treated Tg mice (Fig. 3B). We also observed upregulated expression of *Cd137*and *Tmem26*, two well-known beige progenitor markers^19,31,39^, in the ingWAT of DT-treated Tg mice (Fig. 3C). A flow cytometry analysis further confirmed that CD137^+^ beige progenitors were significantly increased in cold-stimulated Tg mice compared with littermate control mice (Fig. 3D and Fig. S7), indicating that the partial depletion of CD206^+^ M2-like macrophages might regulate the proliferation of beige progenitors. To confirm the proliferating nature of the beige progenitors, we performed an immunofluorescence analysis of ingWAT. Confocal imaging of ingWAT showed that CD137- and UCP1^+^ cells (stained with anti-CD137 and anti-UCP1 antibodies, respectively) were increased in CD206-depleted mice compared with their WT littermates (Fig. 4A), showing that the number of beige progenitors was increased after the partial depletion of CD206^+^ M2-like macrophages. Furthermore, we also observed an increase in CD137- and Ki-67^+^ cells (stained with anti-CD137 and anti-Ki-67 antibodies, respectively) in CD206-depleted mice (Fig. 4B), suggesting that the partial depletion of CD206^+^ M2-like macrophages enhances the proliferation of beige progenitors in the ingWAT of mice under cold exposure.

## Discussion

We examined the role of CD206^+^ M2-like macrophages in the cold-induced browning of ingWAT using genetically engineered CD206DTR mice, in which CD206^+^ M2-like macrophages were partially depleted. Partial depletion of CD206^+^ M2-like macrophages upregulated the expression of *Ucp1* and other browning-related marker genes in ingWAT after cold exposure, which was confirmed by immunohistochemical analysis (showing enhanced expression of UCP1). Gene expression and immunohistochemical analysis revealed that proliferating cells are increased in Tg mice. Immunofluorescence and flow cytometry analysis further confirmed that beige progenitors are enhanced in the ingWAT of cold-induced Tg mice, suggesting that depletion of CD206^+^ M2-like macrophages promotes browning of ingWAT *via* proliferation of beige progenitors. In summary, we found that CD206^+^ M2-like macrophages inhibit the proliferation of beige progenitors and that the partial depletion of CD206^+^ M2-like macrophages releases this inhibition, thereby enhancing browning and insulin sensitivity.

We confirmed partial depletion of CD206^+^ M2-like macrophages by gene expression studies, showing that except for *Il10*, the expression of M2 marker genes was reduced in both ingWAT and BAT. However, DT treatment upregulated the expression of some M1 marker genes aside from *Nos2*, which might affect the enhanced browning in ingWAT of Tg mice in response to cold. We observed upregulated expression of *Il10* in Tg mice at both RT and cold temperatures, suggesting that *Il10* might be involved in other immune responses to clear apoptotic cells (e.g., CD206^+^ cells).

Whether M2-like macrophages promote or inhibit the browning of ingWAT is debatable. Initially, the polarization of M2-like macrophages was reported to regulate thermogenesis and the browning of ingWAT *via* the synthesis of catecholamines^17^ and by increasing the expression of *Ucp1, Pgc1α* and other browning genes^18–21,40–42^. However, Fischer *et al*. conducted a series of experiments using mouse models with different genetic backgrounds and concluded that both the *in vitro* and the *in vivo* induction of M2-like macrophages have no detectable effect on the synthesis of norepinephrine^22^, suggesting that M2-like macrophages induce browning independently of catecholamine synthesis. On the other hand, other researchers have reported both positive^17,18,21^ and negative^22,23^ data suggesting the involvement of M2-like macrophage-induced catecholamine synthesis in the induction of browning in ingWAT. Recently, the expression levels of genes that control the degradation of catecholamines, including growth differentiation factor 3 and monoamine oxidase A, were reported to be upregulated in ATMs from elderly mice^24^, thus contributing to the impairment of lipolysis in adipose tissue and the sustainment of thermogenesis. In addition, another report showed that subpopulations of macrophages called sympathetic neuron-associated macrophages negatively regulated sympathetic innervations under high-fat diet conditions and that the specific ablation of this subpopulation resulted in the enhancement of BAT recruitment and WAT browning^25^. Although these studies did not mention whether the macrophages were M1 or M2 macrophages, these macrophages were able to inhibit browning by suppressing the activity of sympathetic nerve neurons through the degradation of norepinephrine.

However, how the conditional and partial depletion of M2-like macrophages affects the browning of ingWAT remains unknown. Previously, we reported that CD206^+^ M2-like macrophages play a role in maintaining white adipocyte progenitors in a state of hibernation^12^. Thus, we assumed that CD206^+^ M2-like macrophages somehow inhibit the proliferation of beige progenitors in ingWAT. We further assumed that the depletion of CD206^+^ M2-like macrophages likely promotes insulin sensitivity by increasing the number of beige progenitors upon cold stimulation. In the present study, we examined whether the partial depletion of CD206^+^ M2-like macrophages affected the browning of ingWAT in mice under cold stimulation and demonstrated that this is the case. What, then, are the mechanisms behind this observation? We have two hypotheses: first, CD206^+^ M2-like macrophages may inhibit the transdifferentiation of white adipocytes into beige adipocytes; second, the depletion of CD206^+^ M2-like macrophages may increase the proliferation of beige progenitors. Our data suggest that at least the latter hypothesis is true.

In conclusion, we have shown that the partial depletion of CD206^+^ M2-like macrophages induces the proliferation of beige progenitors in ingWAT, thereby increasing the browning of ingWAT after cold stimulation. Further studies are required to investigate the mechanism by which CD206^+^ M2-like macrophages regulate the browning phenomenon, which has the potential to become an effective therapeutic tool for the prevention and treatment of insulin resistance and obesity.

## Electronic supplementary material

Supplementary Figures 1-7
