# Supplementary material for: Partial depletion of CD206-positive M2-like macrophages induces proliferation of beige progenitors and enhances browning after cold stimulation
Source: Sci Rep. 2018 Oct 1;8:14567. doi: 10.1038/s41598-018-32803-6 (PMC6167387; doi:10.1038/s41598-018-32803-6)
Supplement: Supplementary file 1 — Supplementary Figures 1-7 [file 41598_2018_32803_MOESM1_ESM.pdf]

Partial depletion of CD206-positive M2-like macrophages induces proliferation of beige progenitors and enhances browning after cold stimulation

Authors:

Yoshiko Igarashi[1], Allah Nawaz[1,5,6], Tomonobu Kado[1], Muhammad Bilal[1], Takahide Kuwano[1], Seiji Yamamoto[2], Masakiyo Sasahara[2], Xu Jiuxiang[3], Akiko Inujima[3], Keiichi Koizumi[3], Johji Imura[4], Naotoshi Shibahara[3], Isao Usui[1,7], Shiho Fujisaka[1], Kazuyuki Tobe[1]

Supplementary Figures

Figure S1. Creation of CD206DTR transgenic (Tg) mice

A

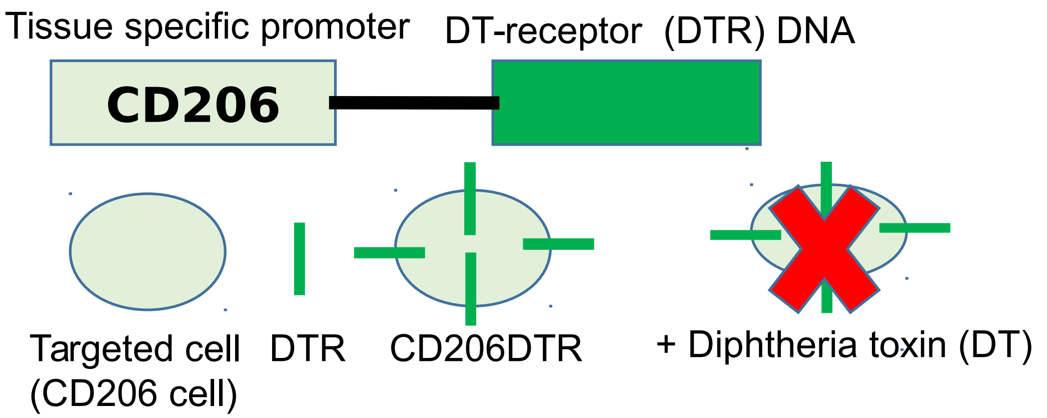

B

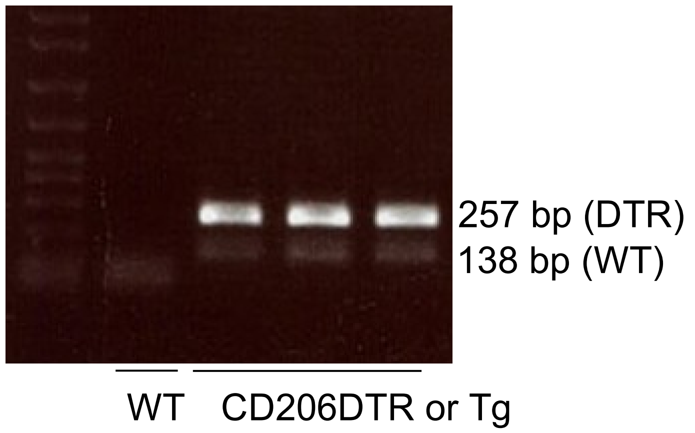

Figure S1. Creation of CD206DTR transgenic (Tg) mice

(A). Creation of CD206DTR transgenic (Tg) mice based on the transgenic expression of diphtheria toxin receptor (DTR) under the control of the CD206-positive promoter to specifically ablate $CD206^{+}$ macrophages. Administration of diphtheria toxin (DT) successfully but partially depleted $CD206^{+}$ macrophages in Tg mice without affecting the overall health of the mice. **(B).** PCR-genotyping analysis showing 257-bp specific sequence of DTR in CD206DTR or Tg mice and 137-bp in WT control mice.

Figure S2. Effect of cold stimulation on BAT and ingWAT

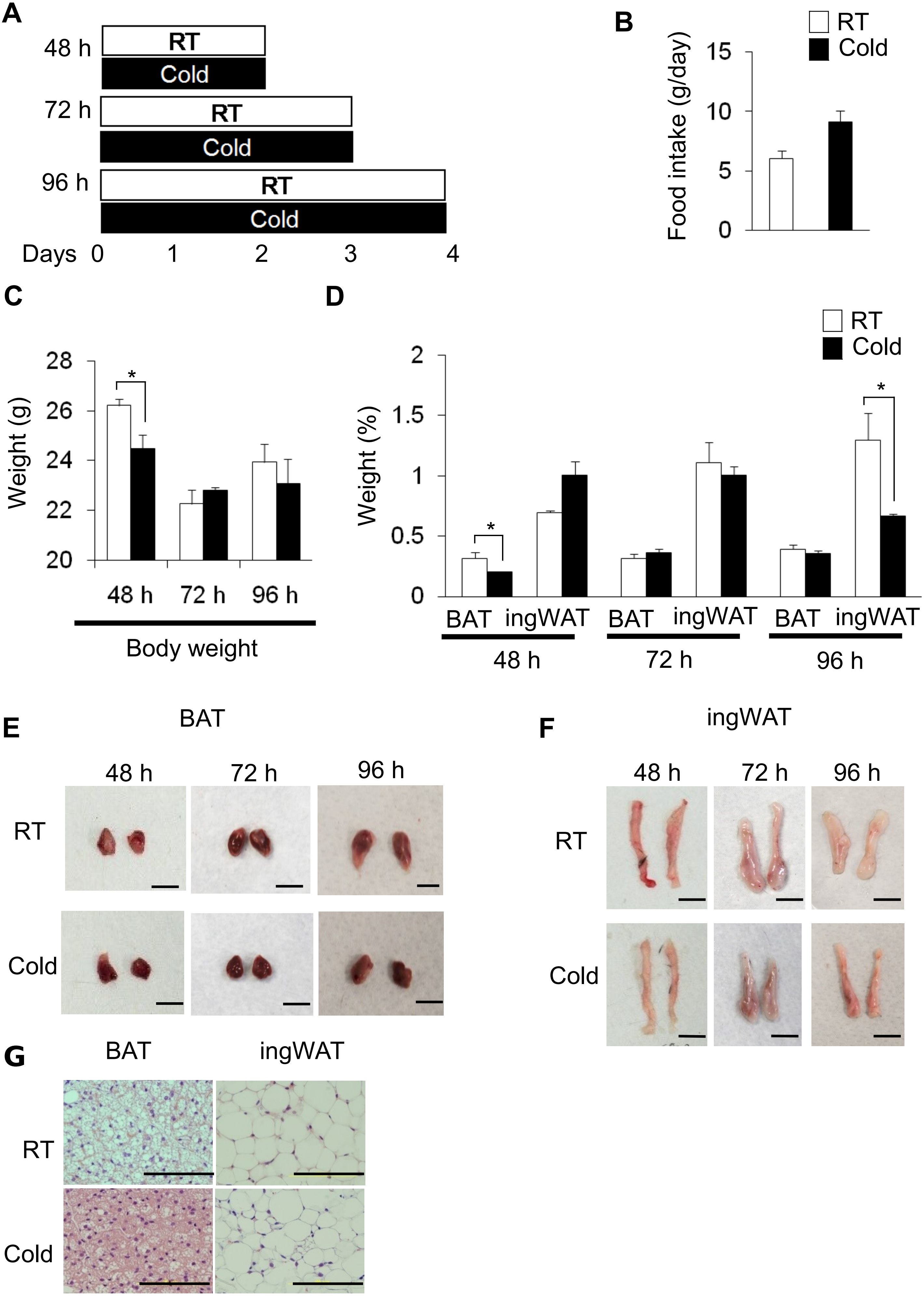

Figure S2. Effect of cold stimulation on BAT and ingWAT
(A). Schematic representation of the experimental time schedule of the cold stimulation. Six groups of WT mice (n = 3 mice per group) were kept under the cold stimulation (6°C) and room temperature (RT) for 48 h, 72 h and 96 h. **(B).** Changes in food intake between the mice maintained at RT and Cold. **(C,D).** Changes in body weight (g), adipose tissue weight (BAT and ingWAT) of RT (white) and cold (black) mice as percentage of body weight (48 h, 72 h and 96 h) (n = 3 mice per group). The data are shown as the means ± SEM. **P* < 0.05. (**E,F).** Representative images of BAT and ingWAT of WT mice (scale bar, 0.5cm). (**G).** Representative images of paraffin sections of BAT and ingWAT of cold stimulated (96 h) WT mice stained with hemotxylin and eosin (H&E). These pictures were taken by using Olympus BX61/DP70, using 40x magnification. Scale bar, 100 μm. (n = 3 mice per group). The data are shown as the means ± SEM. **p* < 0.05, ***p* < 0.01, compared with littermates, as determined using Student's t-test.

Figure S3. Immunohistochemical and gene expression analysis of *UCP1* and other browning genes after cold exposure

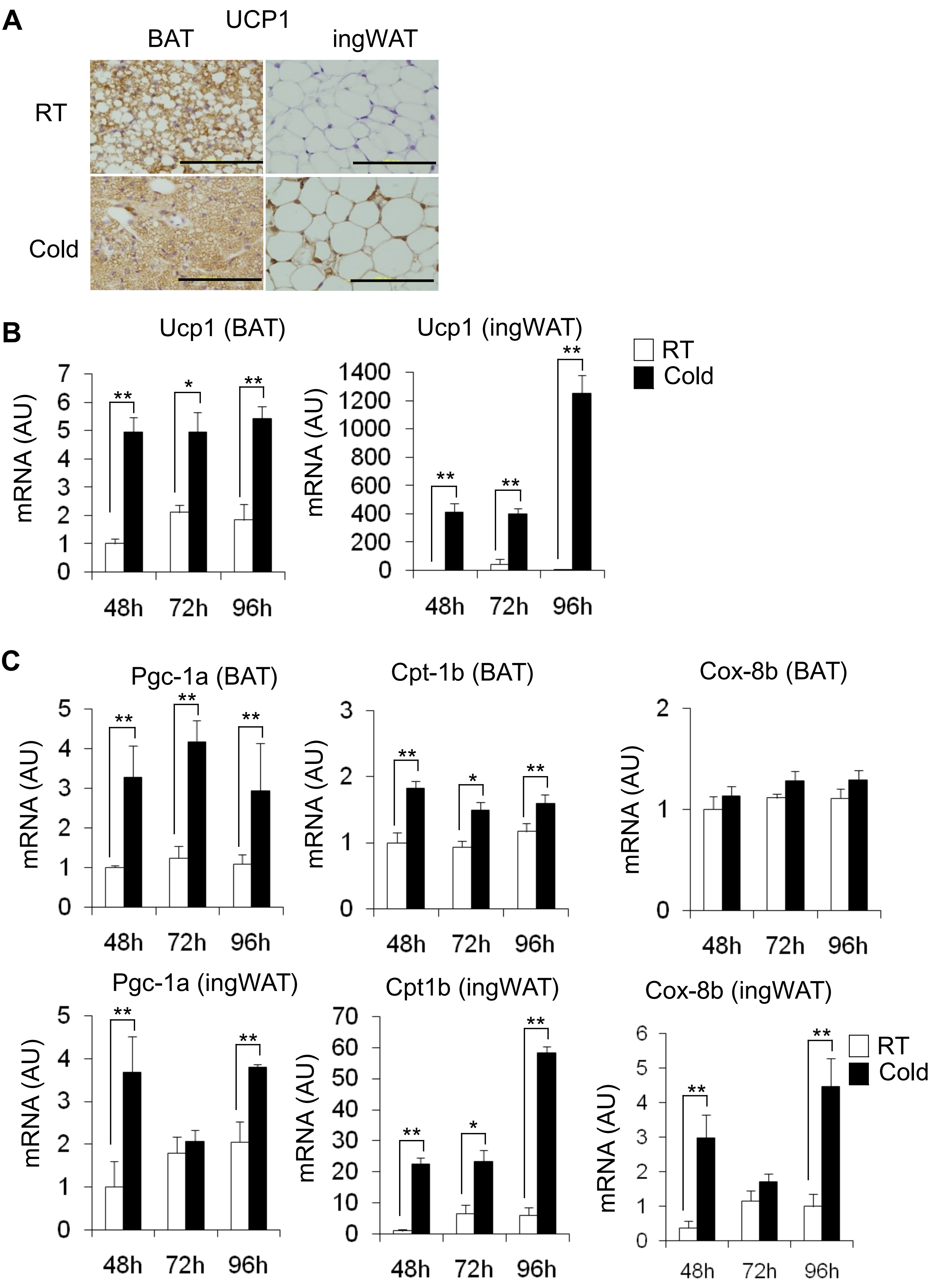

Figure S3. Immunohistochemical and gene expression analysis of *UCP1* and other browning genes after cold exposure

(A). Representative sections of immunostaining of BAT and ingWAT from cold stimulated WT mice compared with control group mice maintained at RT for same duration were stained with anti-UCP1 antibody. Scale bar, 100 μm. (**B,C).** mRNA expression of *Ucp1* and other browning marker genes in the BAT and ingWAT of WT mice maintained at cold and RT (n = 3 mice per group). The data are shown as the means ± SEM. *$p < 0.05$, **$p < 0.01$, compared with littermates, as determined using Student's t-test.

Figure S4. Schematic diagram for DT administration, gene expression analysis and flow cytometry full gating strategy

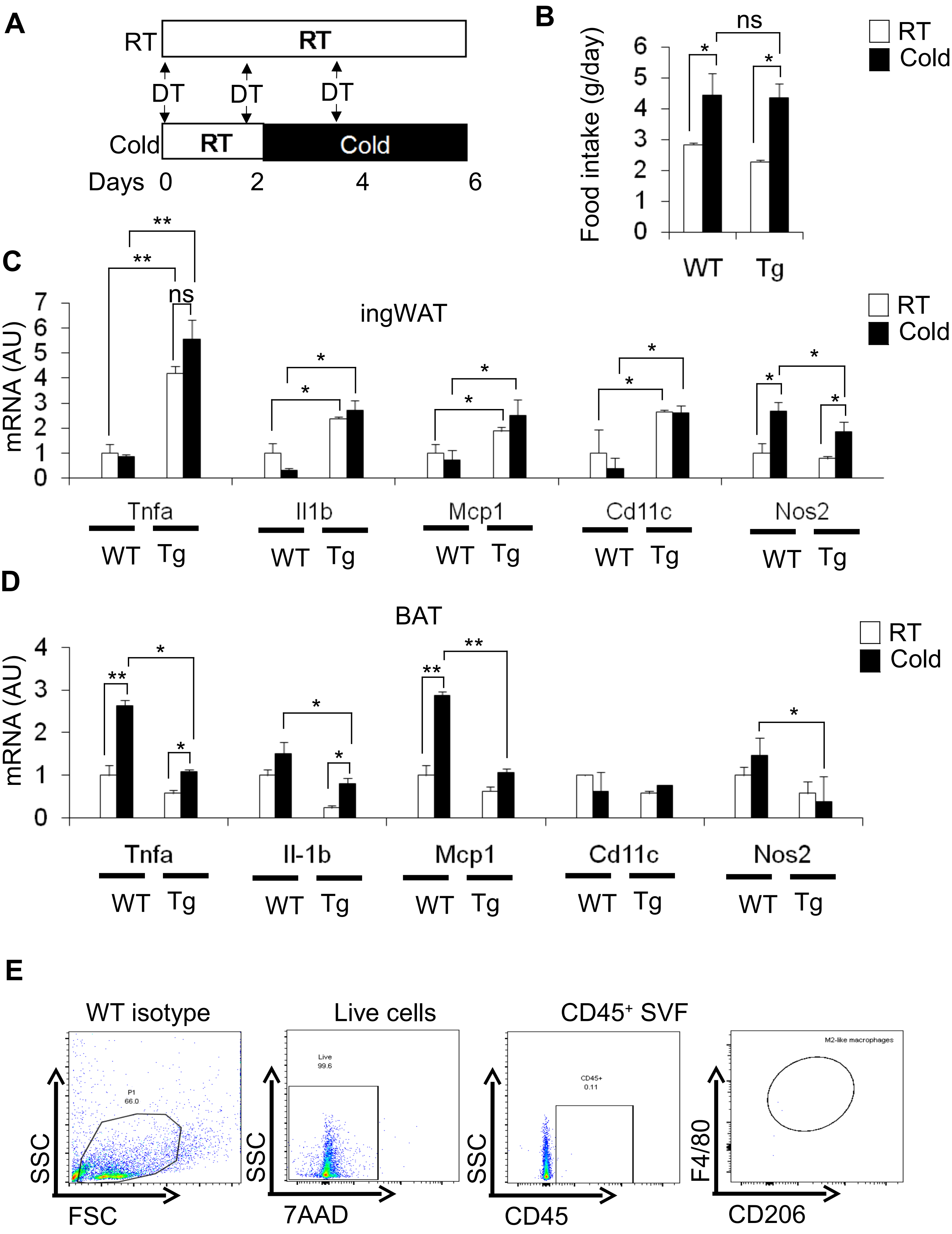

Figure S4. Schematic diagram for DT administration and full gating strategy for flow cytometric analysis of M2-like macrophages
(A). Schematic representation of the experimental time schedule for cold stimulation and DT-treatment in WT and Tg mice. **(B).** Changes in food intake between the WT and Tg mice maintained at RT and Cold. The data are shown as the means ± SEM. *$p < 0.05$, **$p < 0.01$ compared with littermates by Student's t-test. **(C,D).** mRNA expression of M1-like macrophages marker genes in the ingWAT and BAT of cold-stimulated, DT-treated WT and Tg mice compared with their littermate controls at RT (n = 4-6 mice per group). **(E).** Full gating strategy for M2-like macrophages. After removing debris, live ingWAT SVF cells were gated for $CD45^+$ (hematopoietic), and then $CD45^+$ cells are gated for $F4/80^+CD206^+$ double positive population characterized as M2-like macrophages. An isotype was used as a negative control. The experiments were performed using a FACSDiva Version 6.1.2 automated cell analyzer (BD FACSCanto II).

Figure S5. Flow cytometry analysis of BAT

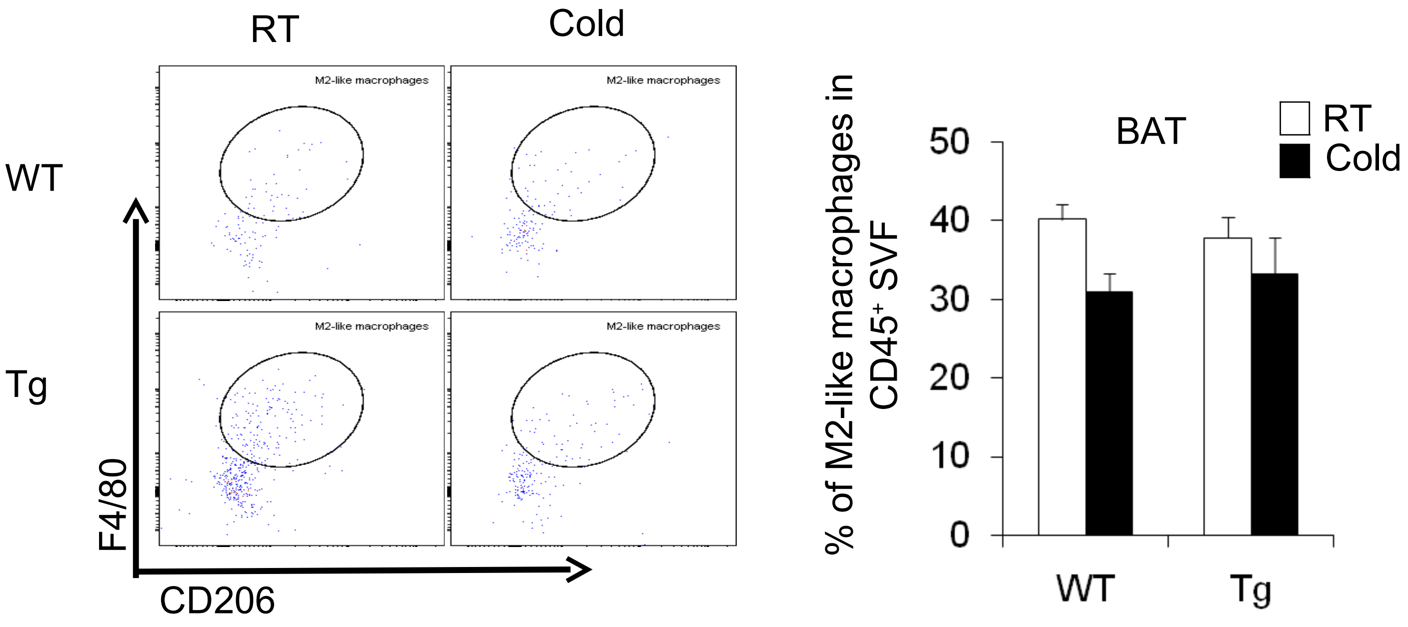

Figure S5. Flow cytometry analysis of BAT

Representative flow cytometric images of BAT from WT and Tg mice (RT vs Cold) (left panel) showing M2-like macrophages. Quantification of M2-like macrophages in $CD45^+$ SVF is given in right panel. After removing debris, live cells were gated for $CD45^+$ (hematopoietic), and then $CD45^+$ cells are gated for $F4/80^+CD206^+$ double positive population characterized as M2-like macrophages. An isotype was used as a negative control (n = 3-4 mice per group). The experiments were performed using a FACSDiva Version 6.1.2 automated cell analyzer (BD FACSCanto II). The data are shown as the means ± SEM. *$p < 0.05$, **$p < 0.01$, compared with littermates, as determined using Student's t-test.

Figure S6. Representative morphological analysis of ingWAT

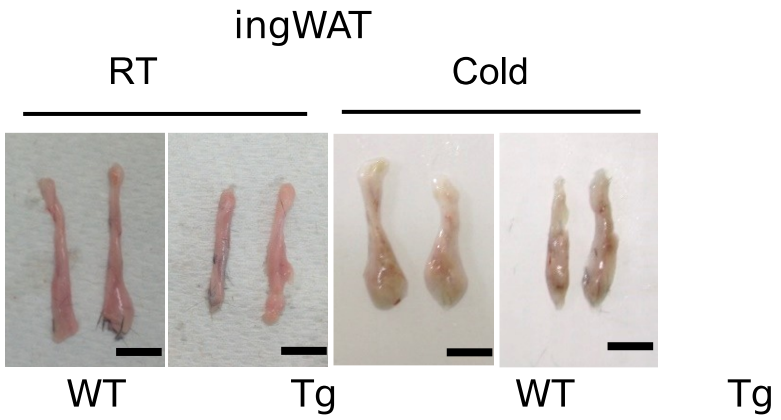

Figure S6. Representative morphological analysis of ingWAT
Representative morphological analysis of ingWAT of DT-treated WT and Tg mice (Scale bar, 0.5 cm).

Figure S7. Flow cytometry analysis of ingWAT

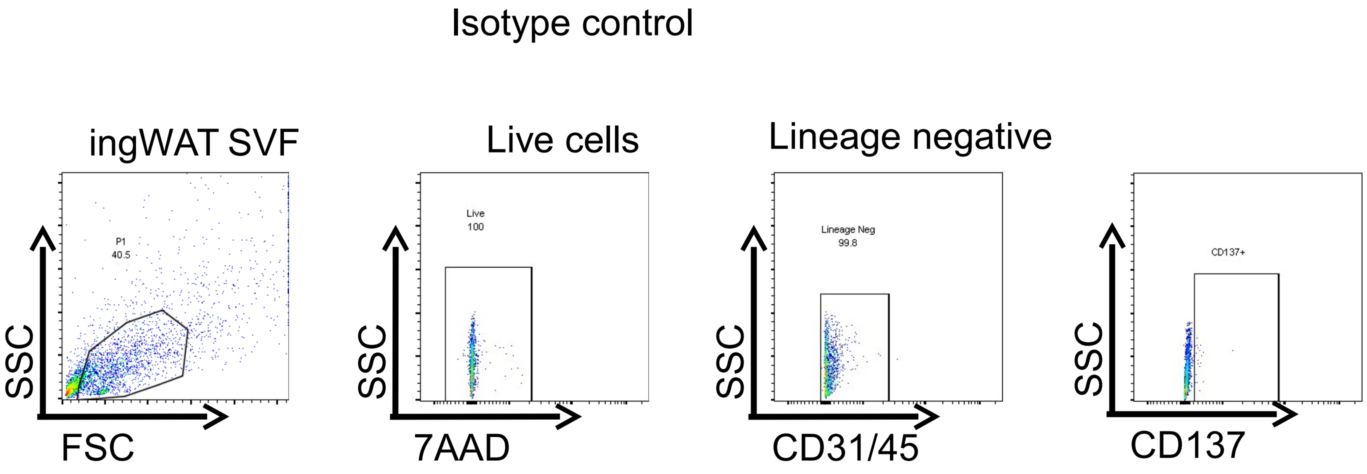

Figure S7. Flow cytometry analysis of ingWAT

Full gating strategy for CD137$^+$ beige progenitors. After removing debris, live ingWAT SVF cells were gated for CD31/CD45$^-$ (endothelial/hematopoietic or lineage negative) population, and then lineage negative population is selected for CD137$^+$ beige progenitors. An isotype was used as a negative control. The experiments were performed using a FACSDiva Version 6.1.2 automated cell analyzer (BD FACSCanto II).
